# Use of a trabecular metal implant in ankle arthrodesis after failed total ankle replacement: A short-term follow-up of 13 patients

**DOI:** 10.3109/17453674.2010.533936

**Published:** 2010-11-26

**Authors:** Anders Henricson, Urban Rydholm

**Affiliations:** ^1^Department of Orthopedic Surgery, Falu Central Hospital, Falun; ^2^Department of Orthopedic Surgery, Lund University Hospital, Lund, Sweden

## Abstract

**Background and purpose:**

Arthrodesis after failed total ankle replacement is complicated and delayed union, nonunion, and shortening of the leg often occur—especially with large bone defects. We investigated the use of a trabecular metal implant and a retrograde intramedullary nail to obtain fusion.

**Patients and methods:**

13 patients with a migrated or loose total ankle implant underwent arthrodesis with the use of a retrograde intramedullary nail through a trabecular metal Tibial Cone. The mean follow-up time was 1.4 (0.6–3.4) years.

**Results:**

At the last examination, 7 patients were pain-free, while 5 had some residual pain but were satisfied with the procedure. 1 patient was dissatisfied and experienced pain and swelling when walking. The implant-bone interfaces showed no radiographic zones or gaps in any patient, indicating union.

**Interpretation:**

The method is a new way of simplifying and overcoming some of the problems of performing arthrodesis after failed total ankle replacement.

Arthrodesis as a salvage procedure after failed total ankle replacement generally shows union rates of 93–100% (Kotnis et al. 2006, Culpan et al. 2007, Schill 2007). However, large bone defects after removal of the prosthesis and debridement of the bony surfaces may prolong the time to bony healing and decrease the union rate (Hopgood et al. 2006). Shortening of the leg is almost inevitable after ankle fusion, and in cases of fusion after failed ankle replacement the shortening tends to be greater and may be a problem.

Trabecular metal (TM; Zimmer, Warsaw, IN) is a new material with several theoretical advantages. It has a porosity and a modulus of elasticity similar to that of trabecular bone (Bobyn et al.1999a). Sufficient bone ingrowth has been shown in animal studies (Bobyn et al. 1999b) and recently in a retrieved human specimen (D'Angelo et al. 2008). The use of TM in hindfoot arthrodesis has been advocated by Frigg et al. (2010). To hopefully overcome the problems associated with delayed union and shortening, we have used a TM Tibial Cone in ankle arthrodesis after removal of a failed prosthesis.

## Patients and methods

We performed ankle arthrodesis using a TM Tibial Cone after failed total ankle replacement in 13 patients. There were 9 STAR prostheses (Waldemar Link, Hamburg, Germany), 2 AES (Biomet, Nimes, France), 1 Mobility (DePuy International, Leeds, UK), and 1 BP prosthesis (Wright Cremasoli, Toulon, France). Failures were due to symptomatic aseptic component loosening or migration with or without periprosthetic osteolysis. The primary indication for total ankle replacement was rheumatoid arthritis in 6 patients, primary osteoarthritis in 3 patients, posttraumatic osteoarthritis in 3 patients, and in 1 patient after an attempt to convert an arthrodesis due to posttraumatic osteoarthritis to a total ankle replacement. The mean time from the index operation to arthrodesis was 7 (2–16) years.

### Surgery

**Figure 1.**
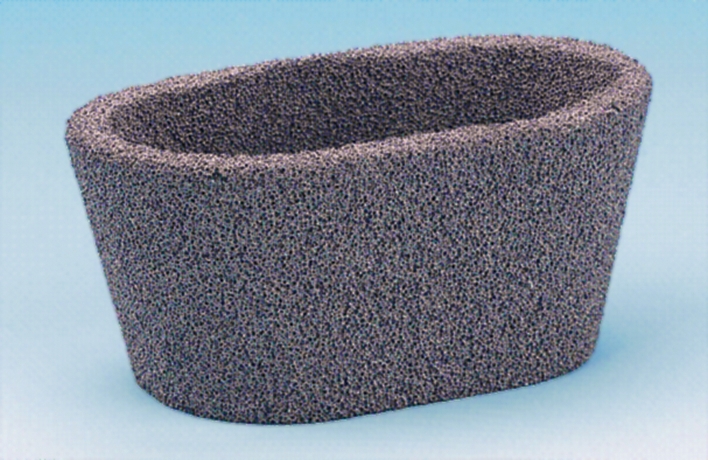
Trabecular metal Tibial Cone.

The original anterior incision was used. The prosthetic components were extracted and the bone surfaces debrided and prepared for a TM Tibial Cone (Zimmer, Warsaw, IN), which is available in 5 different sizes, the smallest being applicable to tibiotalar arthrodesis. The height of the cone is 15 or 30 mm (Figure 1).

**Figure 2.**
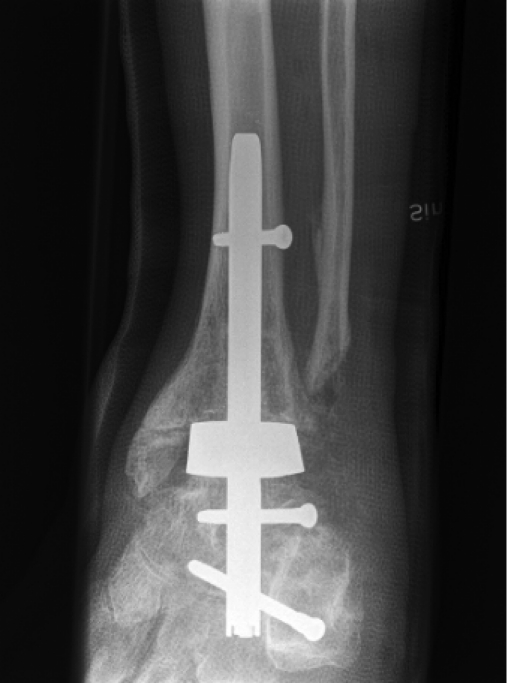
Retrograde intramedullary nailing through a trabecular metal Cone.

The hollow cone was placed between the tibial and talar resection surfaces and the inside was filled with morselized bone, in 9 patients with allograft bone and in 4 with autologous bone from the iliac crest. A retrograde intramedullary nail was placed through the cone and fixed with 2–3 static distal screws and 1 dynamic proximal screw (Figure 2). Further bone graft was then put around the cone.

Partial weight bearing in a below-knee cast was allowed for the first 2–4 weeks, followed by full weight bearing in a cast or a walker boot. The first patients were kept in a cast or boot for 12 weeks, but this period was gradully shortened to 8–10 weeks.

## Results

After a mean follow-up period of 1.4 (0.6–3.4) years, 7 patients were free from pain. 4 patients had some residual pain but were satisfied with the procedure. Slight subtalar pain was experienced by another patient during walking. Furthermore, one patient was dissatisfied with the procedure and suffered from both pain and swelling.

3 secondary procedures were carried out, all with removal of the proximal tibial locking screw. In 2 cases, this was due to pain from the region around the screw and after screw removal the pain gradually disappeared. Another patient sustained a tibial fracture at the level of the proximal screw 2 months postoperatively and the screw had to be removed when the fracture was reduced. The fracture healed in varus malaligment with some residual pain.

Radiographic examinations did not reveal any zones or gaps in the bone-implant interface in any of the patients, and the fusions were thus considered to be united.

## Discussion

After removal of a total ankle replacement, large bone defects often appear. Hopgood et al. (2006) reported that the complication rate and the proportion of nonunions is higher in such cases.

To achieve union in ankle arthrodesis after a failed ankle prosthesis, meticulous debridement of the bony surfaces and extensive bone grafting are necessary. Carlsson et al. (1998) and Anderson et al. (2005) reported that two-thirds of their cases united primarily, a few more after repeated arthrodeses, but in both series 20% of the cases did not unite.

The most common method to preserve leg length is the use of structural (tricortical) autografts (Gabrion et al. 2004, Anderson et al. 2005, Culpan et al. 2007, Schill 2007) or allografts (Anderson et al. 2005). Schill (2007) found union in 14 of 15 cases, Culpan et al. (2007) in 15 of 16 patients, and Gabrion et al. (2004) in 7 of 8 cases with autografts. The 2 patients in the series from Carlsson et al. (1998) with structural allografts did not unite. Successful fusion with preserved leg length in 3 patients respectively was reported by Thomason and Eyres (2008) and Moor et al. (2008) using femoral head allograft and retrograde intramedullary nailing. In an attempt to preserve length, Carlsson (2008) used titanium mesh cages, but none of his 3 cases healed primarily and he advised against this method. Bullens et al. (2010) used retrograde intramedullary nailing through a titanium cage and 1of their 2 cases united primarily.

TM has several favorable properties. Its porosity and modulus of elasticity are close to that of trabecular bone (Bobyn et al. 1999a). After implanting TM acetabular cups in dogs, Bobyn et al. (1999b) found bone ingrowth in all cups, indicating effective biological fixation. Using trabecular implants in carpal joint fusion in an animal study, Adams et al. (2005) found early bone ingrowth and a substantial increase in the strength of the construct over time. Bouchard et al. (2004) reported 1 case of successful use of TM as structural graft in a tarso-metatarsal arthrodesis. TM spacers in hindfoot arthrodeses with 2 years of follow-up led to fusion in all 9 cases in a series by Frigg et al. (2010). These authors had 1 case of failed total ankle replacement, which united with the use a TM spacer, 2 screws, and external fixation. The TM Tibial Cone used in our patients was originally designed for revision surgery in total knee replacement with tibial bone loss. Meneghini et al. (2008) reported good short-term results of this application in 15 revisions of total knee replacement. Long and Scuderi (2009) found signs of stable osseointegration into the cones in 16 knee revisions.

All but 1 of our 13 patients were free from ankle pain already after 6–8 weeks, but since it was difficult to evaluate interface healing radiographically, the first patients were kept in a cast or a boot for a longer period. The ankles continued to be free from pain, but the more proximal pain in one patient treated by extraction of the proximal locking screw and dynamization resulted in subsidence of the implant, probably due to inferior bone quality after multiple surgery. The subsidence was of about the same magnitude as the height of the implant and therefore no effect on the leg length was achieved.

The use of a retrograde intramedullary nail has been suggested by several authors to be the method of choice in fusion after failed total ankle replacements, especially in cases with large bone defects (Anderson et al. 2005, Hopgood et al. 2006, Kotnis et al. 2006). The friction coefficient of trabecular metal is much higher than that of natural bone autografts or allografts, which will enhance the initial stability of this method (Zhang et al. 1999). The intramedullary nail used in our patients has an oval gliding hole for one of the proximal locking screws, which permits a certain amount of compression when axial loading from weight bearing is produced. In all patients, there was an initial subsidence of 4–5 mm corresponding to this gliding hole. The radiographic appearance with no zones around the implants indicates a biologically mature implant-bone interface. Furthermore, since almost all of the patients were free from pain from the ankle at 6–8 weeks postoperatively, we find it justified to assume that there was sufficient stability and provision for bone ongrowth or ingrowth into the implant.

The use of a trabecular metal implant in troublesome ankle fusions after failed total ankle replacement could help restore leg length, provided that the proximal locking screw of the intramedullary nail is retained. Moreover, it appears that since adequate ongrowth or ingrowth of bone probably occurs, the procedure of ankle fusion after failed total ankle replacement is simplified and the need for time consuming extensive bone grafting with painful additional incisions is reduced or unnecessary.

## References

[CIT0001] Adams JE, Zobitz ME, Reach JS, An KN, Lewallen DG, Steinmann SP (2005). Canine carpal joint fusion: a model for four-corner arthrodesis using a porous tantalum implant. J Hand Surg.

[CIT0002] Anderson T, Rydholm U, Besjakov, Montgomery F, Carlsson Å (2005). Tibiocalcaneal fusion using retrograde intramedullary nails as a salvage procedure for failed total ankle prostheses in rheumatoid arthritis. A report of sixteen cases. Foot Ankle Surg.

[CIT0003] Bobyn JD, Stackpool GJ, Hacking SA, Tanzer M, Krygier JJ (1999a). Characteristics of bone ingrowth and interface mechanics of a new porous tantalum biomaterial. J Bone Joint Surg (Br).

[CIT0004] Bobyn JD, Toh KK, Hacking SA, Tanzer M, Krygier JJ (1999b). Tissue response to porous tantalum acetabular cups. J Arthroplasty.

[CIT0005] Bouchard M, Barker LG, Claridge RJ (2004). Tantalum: A structural bone graft option for foot and ankle surgery. Foot Ankle Int.

[CIT0006] Bullens P, Malefijt M, Louwerens JW (2010). Conversion of failed ankle arthroplasy to an arthrodesis. Technique using an arthrodesis nail and a cage filled with morsellized bone graft. Foot Ankle Surg.

[CIT0007] Carlsson Å (2008). Unsuccessful use of a titanium mesh cage in ankle arthrodesis: a report on three cases operated on due to a failed ankle replacement. J Foot Ankle Surg.

[CIT0008] Carlsson ÅS, Montgomery F, Besjakov J (1998). Arthrodesis of the ankle secondary to replacement. Foot Ankle Int.

[CIT0009] Culpan P, Le Strat V, Piriou P, Judet T (2007). Arthrodesis after failed total ankle replacement. J Bone Joint Surg (Br).

[CIT0010] D'Angelo F, Murena L, Campagnolo M, Zatti G, Cherubino P (2008). Analysis of bone ingrowth on a tantalum cup. Indian J Orthop.

[CIT0011] Frigg A, Dougall H, Boyd S, Nigg B (2010). Can porous tantalum be used to achieve ankle and subtalar arthrodesis?. Clin Orthop.

[CIT0012] Gabrion A, Jardé O, Havet E, Mertl P, Olory B, de Laestang M (2004). Ankle arthrodesis after failure of a total ankle prosthesis. Eight cases. Rev Chir Orthop Reparatrice Appar Mot.

[CIT0013] Hopgood P, Kumar R, Wood PLR (2006). Ankle arthrodesis for failed total ankle replacement. J Bone Joint Surg (Br).

[CIT0014] Kotnis R, Pasapula C, Anwar F, Cooke PH, Sharp RJ (2006). The management of failed ankle replacement. J Bone Joint Surg (Br).

[CIT0015] Long WJ, Scuderi GR (2009). Porous tantalum cones for large metaphyseal tibial defects in revision total knee arthroplasty. A minimum 2-year follow-up. J Arthroplasty.

[CIT0016] Meneghini MR, Lewallen DG, Hanssen AD (2008). Use of porous tantalum metaphyseal cones for severe tibial bone loss during revision total knee replacement. J Bone Joint Surg (Am).

[CIT0017] Moor BK, Klammer G, Maquieira GJ, Espinosa N (2008). Salvage arthrodesis after failed total ankle replacement: reconstruction with structural allograft and intramedullary nail. Tech Foot Ankle Surg.

[CIT0018] Schill S (2007). Ankle arthrodesis with interposition graft as a salvage procedure after total ankle replacement. Oper Orthop Traumatol.

[CIT0019] Thomason K, Eyres KS (2008). A technique of fusion for failed total replacement of the ankle. Tibio-allograft-calcaneal fusion with a locked retrograde intrmedullary nail. J Bone Joint Surg (Br).

[CIT0020] Zhang Y, Ahn PB, Fitzpatrick DC, Heiner AD, Poggie RA, Brown TD (1999). Interfacial frictional behaviour: cancellous bone, cortical bone. and a novel porous tantalum biomaterial. J Musculoskeletal Res.

